# Global Sentiments Surrounding the COVID-19 Pandemic on Twitter: Analysis of Twitter Trends

**DOI:** 10.2196/19447

**Published:** 2020-05-22

**Authors:** May Oo Lwin, Jiahui Lu, Anita Sheldenkar, Peter Johannes Schulz, Wonsun Shin, Raj Gupta, Yinping Yang

**Affiliations:** 1 Wee Kim Wee School of Communication and Information Nanyang Technological University Singapore Singapore; 2 School of New Media and Communication Tianjin University Tianjin China; 3 Institute of Communication and Health University of Lugano Lugano Switzerland; 4 School of Culture and Communication University of Melbourne Melbourne Australia; 5 Institute of High Performance Computing Agency for Science, Technology and Research Singapore Singapore

**Keywords:** COVID-19, Twitter, pandemic, social sentiments, emotions, infodemic

## Abstract

**Background:**

With the World Health Organization’s pandemic declaration and government-initiated actions against coronavirus disease (COVID-19), sentiments surrounding COVID-19 have evolved rapidly.

**Objective:**

This study aimed to examine worldwide trends of four emotions—fear, anger, sadness, and joy—and the narratives underlying those emotions during the COVID-19 pandemic.

**Methods:**

Over 20 million social media twitter posts made during the early phases of the COVID-19 outbreak from January 28 to April 9, 2020, were collected using “wuhan,” “corona,” “nCov,” and “covid” as search keywords.

**Results:**

Public emotions shifted strongly from fear to anger over the course of the pandemic, while sadness and joy also surfaced. Findings from word clouds suggest that fears around shortages of COVID-19 tests and medical supplies became increasingly widespread discussion points. Anger shifted from xenophobia at the beginning of the pandemic to discourse around the stay-at-home notices. Sadness was highlighted by the topics of losing friends and family members, while topics related to joy included words of gratitude and good health.

**Conclusions:**

Overall, global COVID-19 sentiments have shown rapid evolutions within just the span of a few weeks. Findings suggest that emotion-driven collective issues around shared public distress experiences of the COVID-19 pandemic are developing and include large-scale social isolation and the loss of human lives. The steady rise of societal concerns indicated by negative emotions needs to be monitored and controlled by complementing regular crisis communication with strategic public health communication that aims to balance public psychological wellbeing.

## Introduction

The coronavirus disease (COVID-19) pandemic has infected individuals in more than 200 countries and resulted in many deaths [1]. With the World Health Organization’s (WHO’s) pandemic declaration and government-initiated actions against the disease, sentiments about COVID-19 are rapidly evolving. In the past decade, social media analytic tools have been utilized to monitor public sentiments and communication patterns of public health emergencies like the Ebola and Zika epidemics. Although many studies have investigated general sentiment valences and discourse topics [2,3], specific emotions have been found to be more closely linked to psychological processes and behaviors than the overall positive and negative valences [4]. Therefore, we postulate that distinct emotions emerging from social media and their underlying narratives are highly relevant to the current COVID-19 crisis and can provide actionable insights into the efficacy of public health messaging.

Particularly, we focused on four emotions: fear, anger, sadness, and joy. According to Plutchik’s Wheel of Emotions [5], fear-anger and sadness-joy are the basic emotion pairs of opposite experiences. Fear is an unpleasant emotion typically arising from danger or uncertainties caused by circumstances, while anger results from uncertainties caused by others [6]. Sadness is a negative emotion experienced typically after unpleasant circumstances that are out of one’s control, and joy is a positive feeling after pleasant events that are appraised as certain and under control [6]. Investigating the evolution of these four basic emotions can demonstrate the changing dynamics of the public’s experience to the crisis.

In this report, we present the results of Twitter users’ public emotional responses to the pandemic. Trends of the four basic emotions and the narratives underlying those emotions were examined.

## Methods

English tweets related to COVID-19 worldwide posted from January 28 to April 9, 2020, were collected from Twitter’s standard search application programming interface using “wuhan,” “corona,” “nCov,” and “covid” as search keywords. These keywords were selected because they were widely used during the early assessment of the COVID-19 situation. Publicly accessible tweets from any type of account that contained any of the keywords were collected. The underlying emotions of tweets were analyzed using the algorithm *CrystalFeel*, a sentiment analytic technology whose accuracy had been demonstrated (see details and examples in Multimedia Appendix 1) [7]. Pearson *r* correlations were conducted between emotions and date to demonstrate the trends of emotions across time statistically. Word clouds were generated for each of the four emotions based on the top frequent unigrams and bigrams.

## Results

**Figure 1:**
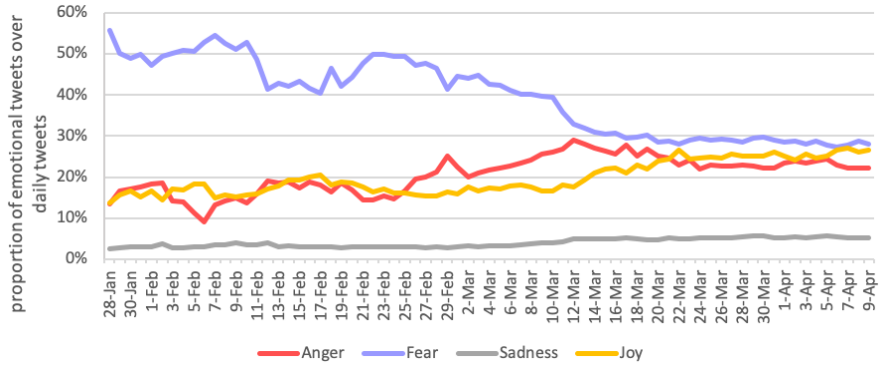
Emotions trends during the early stages of the COVID-19 pandemic.

A total of 20,325,929 tweets were collected, including 7,033,158 unique users from more than 170 countries. The daily proportion of tweets stratified by emotion were plotted across time (Figure 1).

Expectedly, fear was the dominant emotion at the end of January when the disease first surfaced. The prominence of fear gradually dropped to less than 30% of daily tweets in early April as the crisis developed (*r*_71_=–0.92; *P*<.001). In contrast, tweets on anger progressively increased from late January to early March, peaking at 29% on March 12, a day after the pandemic declaration by the WHO. Tweets on anger slightly decreased since then, but remains at a relatively high level (*r*_71_=0.75; *P*<.001). Coinciding with the decrease of tweets on both fear and anger after the pandemic announcement, tweets on sadness, although proportionally lower than those of the other emotions, doubled since the WHO declaration (*r*_71_=0.88; *P*<.001). Similarly, tweets on joy, suggesting a sense of pride, gratitude, hope, and happiness [7], also increased (*r*_71_=0.86; *P*<.001).

Further analyses using word clouds suggest that narratives underlying those emotions evolved as the pandemic developed (Multimedia Appendix 2). In late January, fear was possibly related to the emergence of COVID-19 and its unknown nature, causing uncertainty about containment and spread, indicated by words such as “first case” and “outbreak.” However, as the pandemic escalated, the narratives suggested fear about shortages of COVID-19 tests and medical supplies indicated by words such as “test shortages” and “uncounted.” The anger word clouds suggest xenophobia at the beginning of the pandemic when the disease was predominantly localized to China and Asia, indicated by words such as “racist” and “Chinese people.” Anger then shifted to discourse around isolation fatigue that can occur from social seclusion, indicated by words such as “stay home” and several swear words. Narratives of recent sadness surrounding the topics of losing friends and family members are surfacing, with words relating to “loved one” and “passed away,” highlighting potential social concerns arising from personal traumatic experiences of the pandemic. The world has also seen a concurrent increase in the sense of joy encompassing hope, gratitude, and human resilience with words such as “Thank,” “good news,” and “feel good.”

## Discussion

Our initial findings suggest that global online discourse is swiftly evolving. The discourse is driven by shared public experiences of the COVID-19 pandemic, including large-scale social isolation and the loss of human lives. Although existing studies have demonstrated the immediate psychological reactions to COVID-19 [8,9], our study is the first to demonstrate the evolution of responses across time.

Our findings reveal that negative emotions are dominant during the COVID-19 pandemic, supporting the recent call for action to maintain the public’s mental wellbeing for this unprecedented crisis [10]. Negative emotions such as anger and sadness, which are increasing, need to be heeded and counterbalanced by complementing regular crisis communication with strategic public health communication that aims to balance public psychological wellbeing [2]. If such overbearing public emotions are not addressed, there is potential for the emergence of unintended outcomes such as breeding mistrust in the handling of the disease and a belief in online falsehoods that could hinder the ongoing control of the disease [11,12].

Although the data, collected from Twitter's standard application programming interface, looks at only public tweets surrounding the four selected keywords, the estimation is appropriate for the public discourse surrounding the pandemic at present that abides to ethnical guidelines. Future studies should further investigate sentiments by examining specific countries and expanding the scope to include other media platforms such as Facebook and Weibo.

## Appendix

Multimedia Appendix 1 Extended details on methods and data analysis.

Multimedia Appendix 2 Narratives of emotions during the COVID-19 pandemic.

## Abbreviations

COVID-19: coronavirus disease
WHO: World Health Organization

## References

[1] World Health Organization.

[2] Lwin MO, Lu J, Sheldenkar A, Schulz PJ (2018). Strategic Uses of Facebook in Zika Outbreak Communication: Implications for the Crisis and Emergency Risk Communication Model. Int J Environ Res Public Health.

[3] Zhang EX, Yang Y, Di Shang R, Simons JJP, Quek BK, Yin XF, See W, Oh OSH, Nandar KST, Ling VRY, Chan PP, Wang Z, Goh RSM, James L, Tey JSH (2015). Leveraging social networking sites for disease surveillance and public sensing: the case of the 2013 avian influenza A(H7N9) outbreak in China. Western Pac Surveill Response J.

[4] Moors A (2017). The Integrated Theory of Emotional Behavior Follows a Radically Goal-Directed Approach. Psychological Inquiry.

[5] Plutchik R, Plutchik R, Kellerman H (1980). A general psychoevolutionary theory of emotion. Emotion: Theory, Research, and Experience.

[6] Roseman IJ (1996). Appraisal Determinants of Emotions: Constructing a More Accurate and Comprehensive Theory. Cognition & Emotion.

[7] Gupta R, Yang Y (2018). CrystalFeel at SemEval-2018 Task 1: Understanding and Detecting Emotion Intensity using Affective Lexicons. ACL Anthology.

[8] Wang C, Pan R, Wan X, Tan Y, Xu L, Ho CS, Ho RC (2020). Immediate Psychological Responses and Associated Factors during the Initial Stage of the 2019 Coronavirus Disease (COVID-19) Epidemic among the General Population in China. Int J Environ Res Public Health.

[9] Li S, Wang Y, Xue J, Zhao N, Zhu T (2020). The Impact of COVID-19 Epidemic Declaration on Psychological Consequences: A Study on Active Weibo Users. Int J Environ Res Public Health.

[10] Holmes EA, O'Connor RC, Perry VH, Tracey I, Wessely S, Arseneault L, Ballard C, Christensen H, Cohen Silver R, Everall I, Ford T, John A, Kabir T, King K, Madan I, Michie S, Przybylski AK, Shafran R, Sweeney A, Worthman CM, Yardley L, Cowan K, Cope C, Hotopf M, Bullmore E (2020). Multidisciplinary research priorities for the COVID-19 pandemic: a call for action for mental health science. Lancet Psychiatry.

[11] Bavel JJV, Baicker K, Boggio PS, Capraro V, Cichocka A, Cikara M, Crockett MJ, Crum AJ, Douglas KM, Druckman JN, Drury J, Dube O, Ellemers N, Finkel EJ, Fowler JH, Gelfand M, Han S, Haslam SA, Jetten J, Kitayama S, Mobbs D, Napper LE, Packer DJ, Pennycook G, Peters E, Petty RE, Rand DG, Reicher SD, Schnall S, Shariff A, Skitka LJ, Smith SS, Sunstein CR, Tabri N, Tucker JA, Linden SVD, Lange PV, Weeden KA, Wohl MJA, Zaki J, Zion SR, Willer R (2020). Using social and behavioural science to support COVID-19 pandemic response. Nat Hum Behav.

[12] Duan L, Zhu G (2020). Psychological interventions for people affected by the COVID-19 epidemic. Lancet Psychiatry.

