# Supplementary material for: Global Sentiments Surrounding the COVID-19 Pandemic on Twitter: Analysis of Twitter Trends
Source: JMIR Public Health Surveill. 2020 May 22;6(2):e19447. doi: 10.2196/19447 (PMC7247466; doi:10.2196/19447)
Supplement: Multimedia Appendix 1 [file publichealth_v6i2e19447_app1.docx]

**Extended details on methods and data processing**

A total of 20,325,929 English tweets worldwide related to COVID-19 made from 28 January to 9 April 2020 were collected from Twitter’s standard search application programming interface^[[1]](#footnote-1)^ using “wuhan”, “corona”, “nCov”, and “covid” as search keywords (Table 1). This includes 7,033,158 unique users from more than 170 countries (Figure 1). The single-day peak during this period was 873,236 tweets, which took place on 13 March (Figure 2).

Table A1 Twitter data overview (28 January – 9 April 2020)

| **Keywords** | **Volume / no. of tweets collected** |
| --- | --- |
| wuhan | 1,247,212 |
| corona | 7,761,817 |
| ncov | 167,479 |
| covid | 11,149,421 |
| total | 20,325,929 |

50


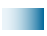


5m


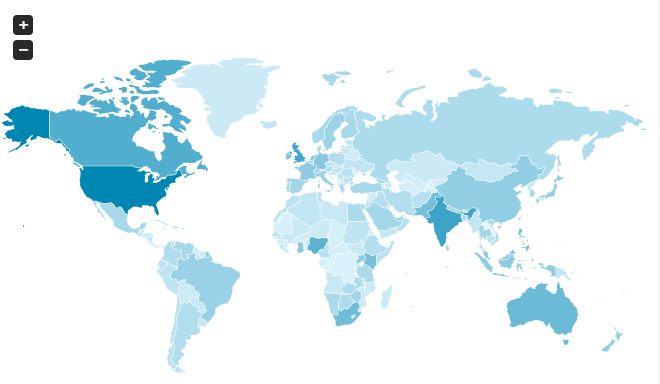


**Figure A1. Spatial distribution of the tweets data**

**Figure A2. Temporal distribution of the tweets data**

The underlying emotions of tweets were analysed using the algorithm CrystalFeel, a sentiment analytic technology^[[2]](#footnote-2)^ whose accuracy had been demonstrated in recent work (Gupta and Yang 2018). CrystalFeel uses features derived from parts-of-speech, n-grams, word embedding, and multiple affective lexicons, and an original in-house developed EI Lexicons to predict the degree of the intensity associated with fear, anger, sadness, and joy in the tweet. The predicted sentiment intensity accuracy had arrived a Pearson correlation coefficient (r) value of .816 on sentiment intensity with out-of-training sample of human annotations, and of .708, .740, .700 and .720 on emotion intensities in predicting joy, anger, fear and sadness as reported in Gupta and Yang (2018).

For our analysis, we used CrystalFeel algorithm’s emotion intensity scores (quantitative values) and converted them into to labels (qualitative values) for more straightforward interpretation, where “fear”, “anger”, “sadness” and “joy” was tagged as each tweet’s dominant “emotion” using the following logic. Table 2 presents a few examples.

emotion_category = "no specific emotion";

if(valence_score > 0.52):

emotion_category = "joy";

elif(valence_score < 0.48):

emotion_category = "anger";

if((fear_score > anger_score) and (fear_score > sadness_score)):

emotion_category = "fear";

elif((sadness_score > anger_score) and (sadness_score > fear_score)):

emotion_category = "sadness";

Table 2. Examples of the tweets data and their corresponding emotion classification results

| **Emotion category** | **Examples** |
| --- | --- |
| anger | Some of you have lost your damn minds....corona has not giving you the right to be a complete asshole! |
| joy | Im continually amazed and grateful for this country. Thank you #armycorpsofengineers! #gratitude #coronavirus |
| fear | i think i have 70 panic attacks every day because of how scared i am for my mom to catch corona |
| sadness | I feel so sad about this Corona virus situation, I feel so...sad. I have cried few times about the death and strugg https://t.co/qdiWv8R1a9 |
| no specific emotion | Up to 10 per cent of recovered virus patients in Wuhan study test positive https://t.co/tNOWpGNDHR https://t.co/d1EskwL6YQ |

**This is a Multimedia Appendix to a full manuscript published in the J Med Internet Res. For full copyright and citation information see** [**http://dx.doi.org/10.2196/jmir.19447**](http://dx.doi.org/10.2196/jmir.19447)**.**

1. <https://developer.twitter.com/en/docs/tweets/search/api-reference/get-search-tweets>. It is useful to note that Twitter standard search API is limited in retrieving *relevant* tweets that match the search keywords, but it does not guarantee the retrieved tweets are exhaustive or complete. [↑](#footnote-ref-1)
2. [www.crystalfeel.socialanalyticsplus.net](http://www.crystalfeel.socialanalyticsplus.net) [↑](#footnote-ref-2)
