# Supplementary material for: Global Sentiments Surrounding the COVID-19 Pandemic on Twitter: Analysis of Twitter Trends
Source: JMIR Public Health Surveill. 2020 May 22;6(2):e19447. doi: 10.2196/19447 (PMC7247466; doi:10.2196/19447)
Supplement: Multimedia Appendix 2 [file publichealth_v6i2e19447_app2.docx]

**Narratives of emotions during the COVID-19 pandemic**

| Emotions | 30 January to 1February | 6 April to 9 April |
| --- | --- | --- |
| Fear | *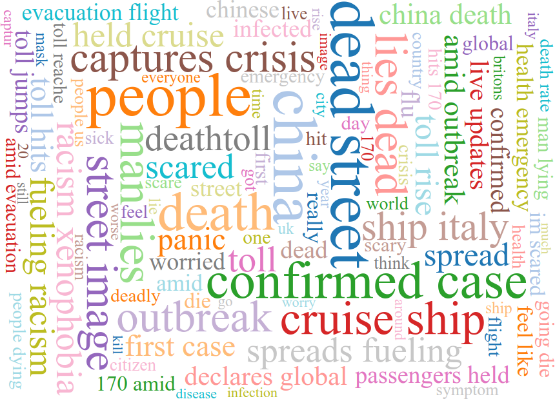* | 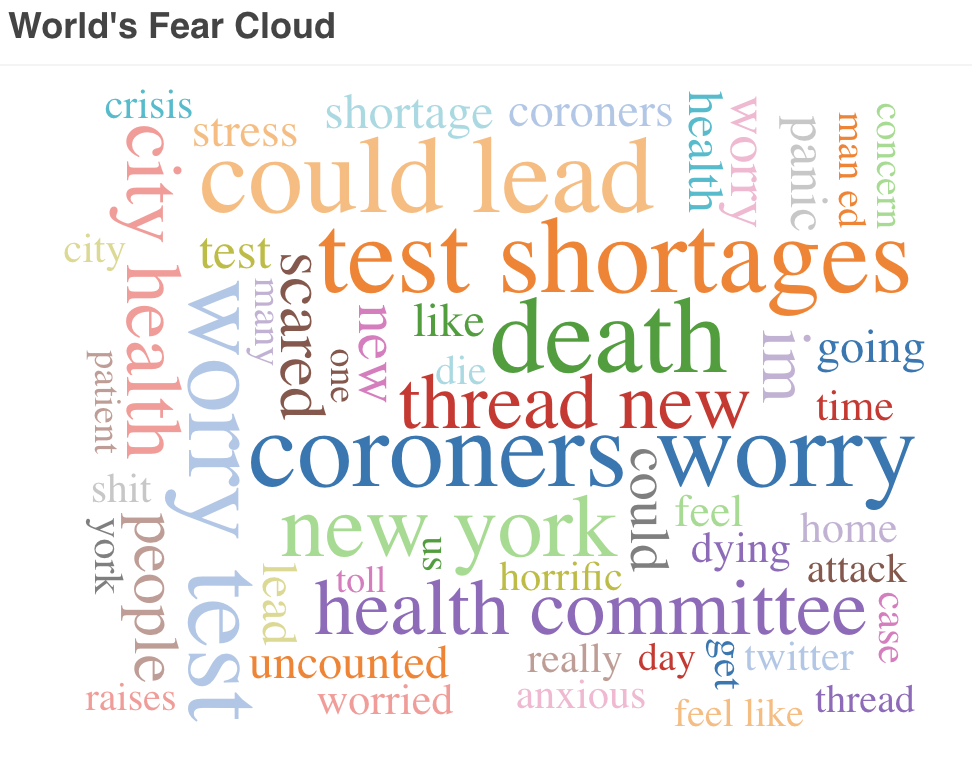 |
| Anger | *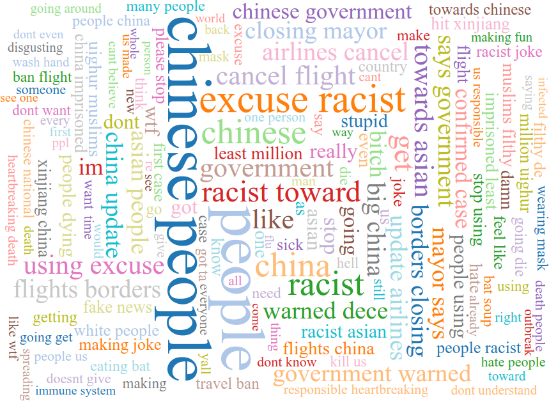* | 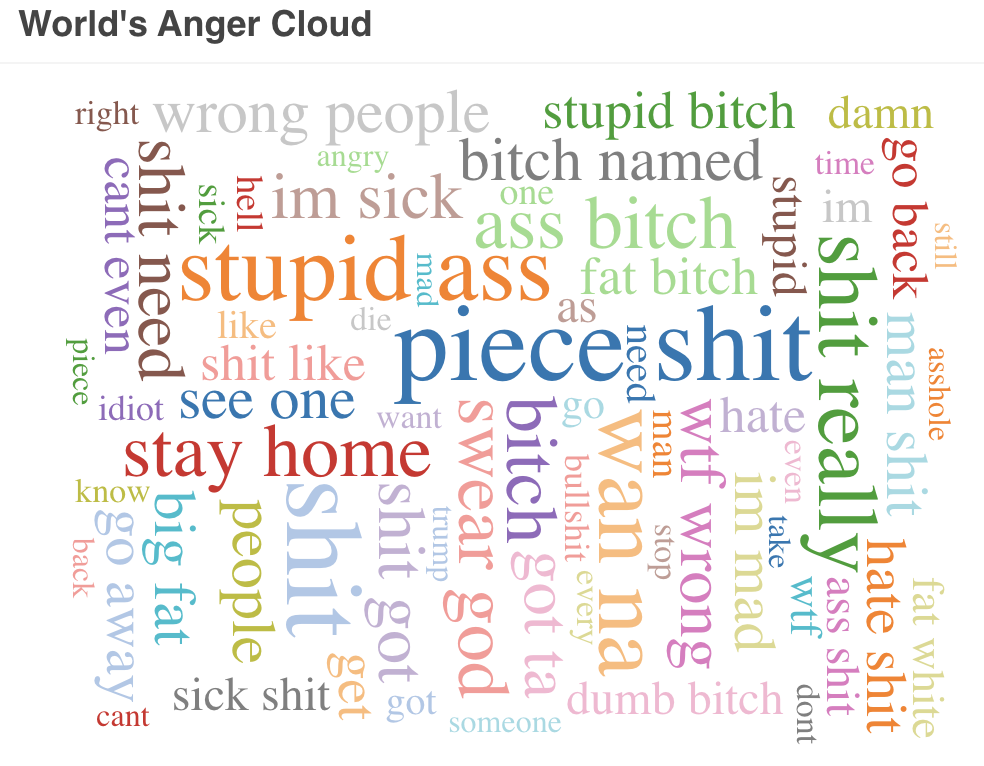 |
| Sadness | *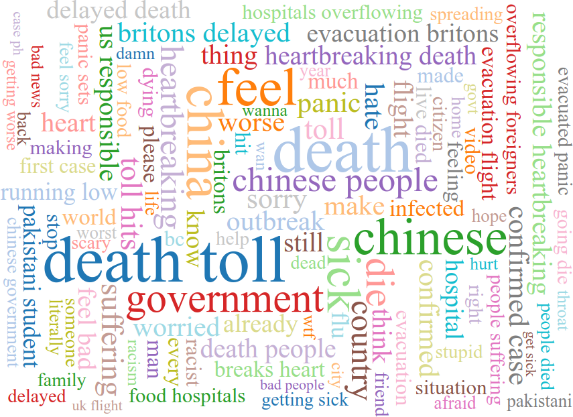* | 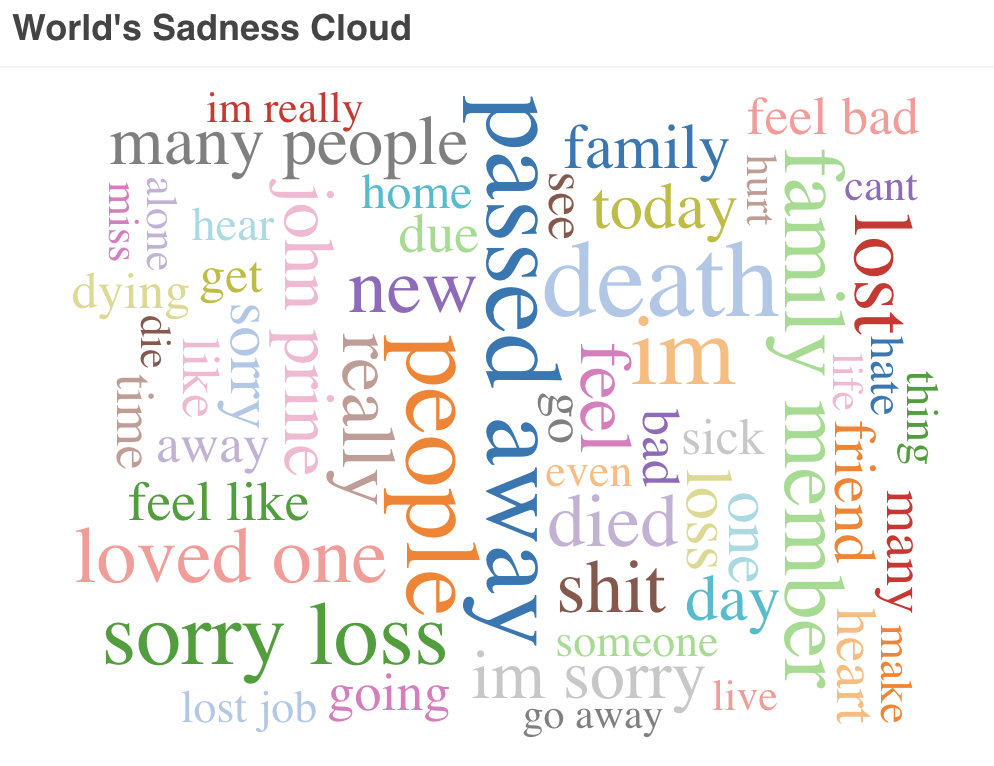 |
| Joy | *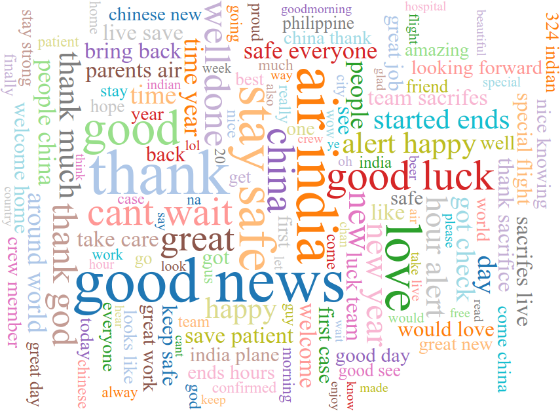* | 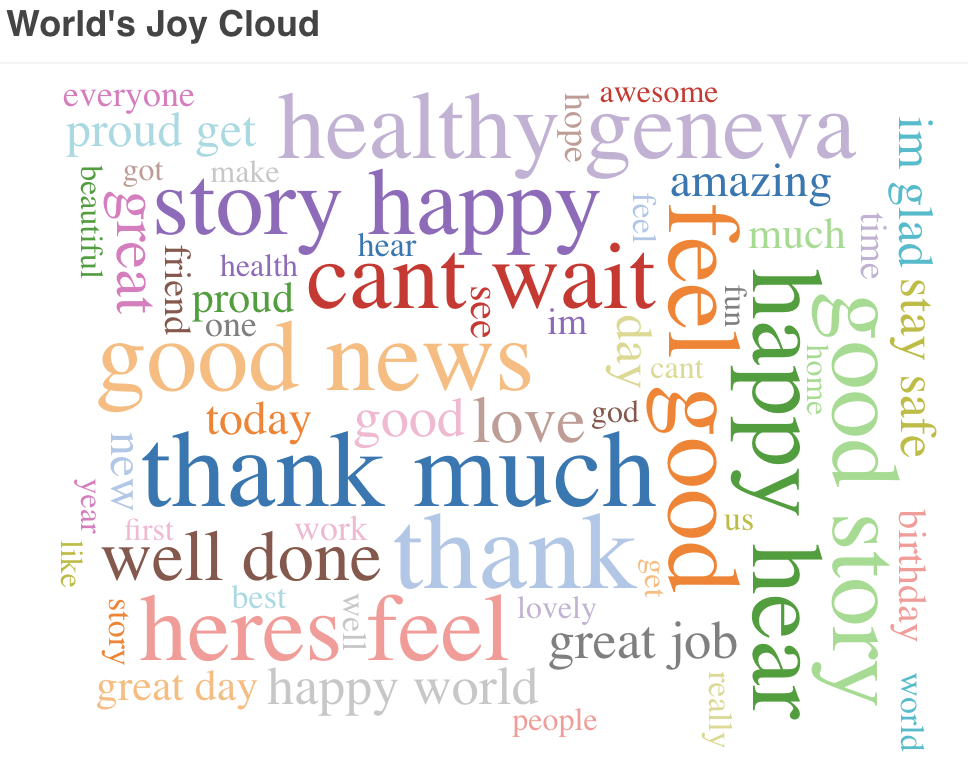 |
